# Hyperkalemia: Pharmacotherapies and Clinical Considerations

**DOI:** 10.7759/cureus.52994

**Published:** 2024-01-26

**Authors:** Evan S Sinnathamby, Kelly T Banh, William T Barham, Tyler D Hernandez, Audrey J De Witt, Danielle M Wenger, Vincent G Klapper, David McGregor, Antonella Paladini, Shahab Ahmadzadeh, Sahar Shekoohi, Alan D Kaye, Giustino Varrassi

**Affiliations:** 1 Medicine, Louisiana State University Health Sciences Center, New Orleans, USA; 2 Medicine, Louisiana State University Health Sciences Center, Shreveport, USA; 3 Medicine, University of Arizona College of Medicine - Phoenix, Phoenix, USA; 4 Internal Medicine, Louisiana State University Health Sciences Center, Shreveport, USA; 5 Anesthesiology, Louisiana State University Health Sciences Center, Shreveport, USA; 6 Life, Health and Environmental Sciences (MESVA), University of L'Aquila, L'Aquila, ITA; 7 Pain Medicine, Paolo Procacci Foundation, Rome, ITA

**Keywords:** medication, resins, patiromer, hyperkalemia, potassium

## Abstract

Hyperkalemia has been defined as a condition where a serum potassium level is >5.5 mmol/l. It is associated with fatal dysrhythmias and muscular dysfunction. Certain medical conditions, such as chronic kidney disease (CKD), diabetes mellitus, and others, can lead to hyperkalemia. Many of the signs of hyperkalemia are nonspecific. A history and physical examination can be beneficial in the diagnosis of the condition. In this regard, certain characteristic electrocardiogram findings are associated with hyperkalemia along with laboratory potassium levels. In acute and potentially lethal conditions, hyperkalemia treatments include glucose and insulin, bicarbonate, calcium gluconate, beta-2 agonists, hyperventilation, and dialysis. There are several drugs, both old and new, that can additionally aid in the reduction of serum potassium levels. The present investigation evaluated some of these different drugs, including sodium polystyrene sulfonate (SPS), sodium zirconium cyclosilicate (SZC), and patiromer. These drugs each have increased selectivity for potassium and work primarily in the gastrointestinal (GI) tract. Each of these medications has unique benefits and contraindications. Clinicians must be aware of these medications when managing patients with hyperkalemia.

## Introduction and background

Hyperkalemia is a potentially life-threatening metabolic disorder where the kidneys cannot excrete potassium efficiently or there is an inability of the body to move potassium into cells from circulation [1]. Hyperkalemia can also be due to a combination of both factors. In these cases, potassium levels can rise to >5.5 mmol/l and are associated with morbidity and mortality.

Commonly, medications that alter potassium homeostasis are the triggers of acute hyperkalemia. Illness and low water intake can also cause hyperkalemia. For example, diabetes mellitus can cause nephropathy and lead to hyporeninemic hypoaldosteronism. Aldosterone is needed to retain sodium from collecting ducts and excrete potassium. If there is low aldosterone, potassium levels in the body will rise. Electrocardiographic changes can be seen in hyperkalemia. In initial electrocardiogram (ECG) changes with hyperkalemia, T waves become narrow, pointed, and tall; these changes are seen in all leads on the ECG. As the hyperkalemia progresses, other ECG abnormalities may occur, such as decreased P wave height, PR prolongation, a widened QRS, and, eventually, the characteristic sinusoidal pattern [2, 3]. In addition to cardiogenic dysfunction, muscular dysfunction, which may lead to weakness and flaccid paralysis, may also be seen [4]. Other symptoms of hyperkalemia are typically nonspecific and include nausea, vomiting, chest tightness, paresthesia, and palpitations [5]. Urine potassium, creatinine, and osmolarity should be obtained in determining the cause of hyperkalemia. Intravenous calcium gluconate is effective in the acute management of hyperkalemia by preventing cardiac dysfunction, but it does not lower serum potassium levels [1]. Serum potassium levels can be lowered acutely by using intravenous insulin and glucose or nebulized beta-2 agonists.

There are several major risk factors for developing hyperkalemia. One of the most significant factors is renal impairment, either by acute kidney injury (AKI) or advanced chronic kidney disease (CKD). Additionally, any acquired or genetic defect in the distal nephron may lead to decreased potassium excretion [6]. Medications can also cause hyperkalemia by altering aldosterone signaling (e.g., non-steroidal anti-inflammatory drugs (NSAIDs) and angiotensin-converting enzyme (ACE) inhibitors), inhibiting sodium reabsorption in the distal convoluted tubule (e.g., potassium-sparing diuretics), or increasing the extracellular translocation of potassium (e.g., somatostatin and isoflurane). Hyperkalemia frequently develops in dialysis-dependent patients [6]. There are several other medications used to lower serum potassium levels. In patients with aldosterone deficiency, fludrocortisone acetate may be used. Sodium polystyrene sulfonate (SPS), a cation exchange resin, is also used to treat hyperkalemia [7]. Potassium-binding agents, like sodium zirconium cyclosilicate (SZC) and patiromer, are novel medications that can be used to lower serum potassium levels. The present investigation, therefore, focuses on pharmacological and clinical factors related to the potassium-binding agents SPS, SZC, and patiromer. The pharmacodynamic and pharmacokinetic factors of each medication, side effects, and contraindications of each drug are described in this review.

## Review

Treating hyperkalemia with SPS

In 1958, the United States Food and Drug Administration (FDA) approved the use of an ion exchange resin, SPS, or Kayexalate, to treat hyperkalemia. Once taken, the resin, which is not systemically absorbed, travels through the gastrointestinal (GI) tract, where it releases sodium ions in exchange for potassium ions and ultimately is excreted via feces. However, SPS is a nonselective ion exchange resin and has the affinity to bind magnesium or calcium ions [8]. It is estimated that for 1 gram of resin, there is a reduction of 1 mEq/L of potassium, but it could be as low as 0.4-0.8 mEq/L given that it is nonselective for potassium. The drug is administered as either an oral suspension dosed anywhere from 15-60 g/day or via rectal enema dosed between 30-60 g/day [9]. Studies have demonstrated that SPS reduces serum potassium in a dose-dependent manner, where increased dosage results in larger reductions of serum potassium. On average, the measured reduction in potassium was 0.99 mEq/dL 10 hours after administration of SPS [10]. The onset of action is inconsistent and varies from one hour to a day, making its use unfavorable in acute settings [11].

Although uncommon, the use of SPS has been associated with severe GI adverse effects such as bleeding, constipation, diarrhea, gastric irritation, perforation, intestinal ischemia, or colonic necrosis. Previously, SPS was co-administered with the laxative sorbitol to reduce constipation and increase potassium excretion. However, this is no longer the standard of care, as the FDA issued a black box warning in 2009 against the use of SPS and sorbitol, given the risk of serious GI complications [8]. The potential for fatal complications limits the use of SPS in the management of hyperkalemia compared to other resin treatment options, especially in patients with a history of intestinal disease and recent surgery leading to reduced GI motility, hypovolemia, and renal insufficiency [12]. Electrolyte abnormalities are rare and minor, but patients using SPS should be monitored, given the risk of hypokalemia, hypomagnesemia, hypocalcemia, and hypernatremia [13]. Given the release of sodium ions into the body and the potential for volume overload, SPS should be used with caution in patients with hypertension and congestive heart failure (Table 1).

**Table 1:** Clinical studies regarding the efficacy and safety of sodium polystyrene sulfonate. CKD: Chronic kidney disease; ED: emergency department; SPS: sodium polystyrene sulfonate; SD: standard deviation; SZC: sodium zirconium cyclosilicate

Author (Year)	Groups studied and intervention	Results and findings	Conclusions
Lepage et al. [14]	Patients with CKD and mild hyperkalemia were administered oral 30 g SPS or placebo once/day for 7 days	The mean difference between groups -1.04 mEq/L with a 95% confidence interval (-1.37 to – 0.71)	SPS was superior to placebo.
Hasara et al. [15]	Patients presenting to the ED with hyperkalemia were administered SPS or SZC	The mean change in serum potassium from baseline to repeat level was -1.1mEq/L for both groups	SPS and SZC administration resulted in a similar reduction of serum potassium.
Nguyen et al. [16]	For Adult patients (18+) admitted for acute hyperkalemia, a dose of patiromer (8.4 g or 16.8 g) or SPS (15 g or 30 g) administered	The mean SD potassium reduction was higher when using SPS compared to patiromer 0.76 (0.63) mEq/L vs 0.32 (0.65) mEq/L, (P = .001)	SPS demonstrated a clinically significant reduction in serum potassium compared to patiromer.

Treatment with SZC

Sodium zirconium cyclosilicate, an orally administered inorganic microporous compound comprised of complex silicon and zirconium ions, shows potential in the management of acute hyperkalemia [17]. Zirconium, a component of SZC, is an element used in dental implants, antiperspirants, and reconstructive implants and is generally accepted as a low-toxicity compound [18]. Sodium zirconium cyclosilicate is thought to act as a phony potassium ions (K+) channel in the gastrointestinal tract, where it exhibits relative selectivity for K+ versus other organic cations, such as calcium (Ca^2^+), magnesium (Mg^2^+), or sodium (Na+) [19]. Sodium zirconium cyclosilicate has no taste or odor and is not systemically absorbed as it traverses the GI tract [20]. Sodium zirconium cyclosilicate exhibits an early and rapid uptake of K+ ions in vitro at acidic, stomach-like proton concentrations (pH 1-3), and this uptake is only increased at higher pH (19). These data are in concordance with in vivo human data, showing a median time of 2.2 hours from serum hyperkalemia (5.6 mEq/L) to serum normokalemia (4.5 mEq/L) [20]. Therefore, it seems that SZC has a delayed effect, not taking up K+ until it reaches the acidic environment of the stomach but only increasing K+ sequestration as it moves into the more basic environment of the duodenum and jejunum. Sodium zirconium cyclosilicate administered three times a day, with meals, for 48 hours has been shown to reduce serum K+ levels to 0.5, 0.5, and 0.7 mmol per liter at 2.5 g, 5 g, and 10 g, respectively, in a phase III randomized control trial [21]. In another phase III study conducted over 57 days in hemodialysis patients, the use of 5 g of SZC had a mean difference in serum K+ of − 0.74 mmol/L relative to the placebo group [22].

In another phase III randomized control trial in patients taking loop diuretics or ACE inhibitors, the use of SZC to maintain normokalemia for less than 12 months has not been associated with renin-angiotensin-aldosterone system inhibitor (RAASi) changes [23]. In CKD patients, SZC may have an additional use as an agent to increase serum bicarbonate; it is thought that this occurs through increased ammonia genesis at lower serum K+ concentrations [24-26]. Additionally, SCZ is hypothesized to increase serum bicarbonate by ammonium ion (NH+) binding, as NH+ is similar in ionic size to K+ [27]. Thus, because SZC facilitates correction for normokalemia and also increases serum bicarbonate, it may be especially useful in CKD and hemodialysis patients [22,25].

Another population that may benefit from reduced K+ levels is heart failure patients, who may not be prescribed ACE inhibitors due to hyperkalemia exacerbation or the risk of new hyperkalemia. Patients with concomitant CKD and heart failure exhibit marked morbidity and mortality, and future research into SZC use in this population could provide a workaround for provider ACE inhibitor prescription reluctance while avoiding potentially severe GI side effects of other accepted medications used to correct hyperkalemia in heart failure patients [28,29]. In previous studies investigating heart failure patients, SZC successfully lowered K+ levels but was associated with an increased risk of edema [20].

Some of the adverse effects of SZC include urinary tract infections (1.1%) and edema (0.9%); however, SZC is generally better tolerated than other K+ reduction medications, such as patiromer, which has been associated with GI discomfort and electrolyte reduction in over 7% of patients in another head-to-head trial [30]. The more rapid removal of K+ with SZC relative to patiromer increases the risk of edema, and this effect is hypothesized to occur via an incidental increase in Na+ absorption [31]. Although SZC has not been studied in patients with impaired GI motility, it may exacerbate this and other GI conditions. However, meta-analyses have failed to find a significant difference in rates of GI issues (nausea, vomiting, diarrhea, and constipation), cardiac disorders, urinary tract infections, and hypokalemia in the SZC cohorts relative to control cohorts [32]. Sodium zirconium cyclosilicate, sold under the trade name Lokelma, is contraindicated in hypokalemic patients as well as patients at risk for motility disorders. Additionally, it is contraindicated in the case of radiographic studies due to its radiopaque appearance in imaging [33]. Because SZC can transitorily increase stomach pH, caution should be exercised when administering SZC at the same time as pH-dependent oral medications [33].

Treating hyperkalemia with patiromer

Patiromer (patiromer sorbitex calcium) is a nonabsorbed, potassium-binding anionic polymer composed of patiromer, a potassium-binding polymer, and a calcium-sorbitol counterion. This chemical structure contributes to the mechanism of action, which involves the preferential binding of potassium in the distal colon, where potassium concentrations are higher than those of sodium, calcium, and magnesium. Following oral administration, the compound's calcium ions are exchanged for potassium ions, reducing serum potassium levels by preventing potassium absorption into the bloodstream and promoting fecal excretion [34,35].

Clinical studies have shown patiromer to be effective in reducing serum potassium levels in patients with hyperkalemia. In a randomized, double-blind, placebo-controlled, parallel-group trial of 105 heart failure cases with a history of hyperkalemia, a significantly reduced mean serum potassium level occurred with patiromer relative to placebo at the end of treatment (between-group difference: −.45 mEq/L (P <0.001)) [35]. In a two-phase study consisting of a four-week, single-group, single-blind initial treatment phase in 219 hyperkalemic CKD patients receiving a RAASi and an eight-week, placebo-controlled, single-blind, randomized withdrawal phase containing 107 patients of the original population, the estimated median change in potassium level to week four of the withdrawal phase showed 0 mmol/L for patiromer as compared to 0.72 mmol/L for placebo (P <0.001) [36]. Safety and efficacy of long-term patiromer use were studied in the AMETHYST-DN randomized clinical trial, which demonstrated that in patients with hyperkalemia and diabetic kidney disease, starting doses of 4.2 to 16.8 g twice daily (8.4-33.6 g/d) resulted in significant decreases in serum potassium levels following four weeks of treatment, lasting through 52 weeks [37]. Real-world experience with patiromer has also shown promising results. A retrospective observational study showed that continuous patiromer exposure was associated with a retained ability to continue RAASi therapy while also resulting in statistically significant reductions in hospital visits and admissions [38].

The recommended initial dose of patiromer is 8.4 g once daily. The dose can be measured based on the patient's serum potassium levels, with a maximum daily dose of 25.2 g. Patiromer should be taken with food for at least three hours before or after other oral medications [39]. The onset of action of patiromer is generally observed within seven hours of the first dose, and potassium level stability is retained for 24 hours after discontinuation [40].

The most common adverse effects of patiromer include GI disturbances such as constipation, diarrhea, nausea, vomiting, and abdominal pain. These side effects are generally reported as mild to moderate in severity and are found to occur in less than 7.2% of patients within the global pharmacovigilance database [39,41]. Hypokalemia has been reported as an adverse effect; however, it is rare and reversible via dose management [36]. In addition to binding to potassium in the colon, patiromer can also bind to magnesium and potentially cause hypomagnesemia [39]. Although hypomagnesemia was only noted in a small proportion of patients, it increases the risk for ventricular arrhythmia. Therefore, electrolyte monitoring and magnesium supplementation should be considered while administering patiromer [39,41,42]. It is recommended that patiromer be avoided in patients with gastrointestinal motility disorders, as it may not work as intended due to poor GI motility and may exacerbate these conditions. Patiromer should also be used with caution in patients with a history of bowel surgery or bowel ischemia, for similar reasons. Due to its delayed onset of action, patiromer should not be used in the emergency treatment of life-threatening hyperkalemia. Patiromer is contraindicated in patients with a history of a hypersensitivity reaction to the drug or any of its components (i.e., xanthan gum) [34].

## Conclusions

Hyperkalemia may be a life-threatening condition. There are certain signs suggestive of hyperkalemia, such as characteristic ECG changes, but many symptoms of hyperkalemia are nonspecific. Certain medications may cause hyperkalemia, and a number of medical conditions, like CKD, can predispose patients to hyperkalemia. Hyperkalemia can be fatal, so it is important to act quickly when it is suspected and diagnosed. Certain medications, like SPS, SZC, and patiromer, can help treat hyperkalemia. The mechanism of SPS involves circulation through the GI tract, where it releases sodium ions in exchange for potassium ions and is ultimately excreted via feces. It is important to note that SPS is nonspecific for potassium ions, and it may exchange other ions like calcium instead. Other resins, like SZC, work similarly to SPS but may have more selectivity for potassium. Finally, patiromer works by selectively binding to potassium in the distal colon, where potassium levels are higher than other ions. All three medications work in the GI tract, so caution must be exercised when prescribing them to patients with GI disorders. Certain patient populations may also benefit from one medication over the other. It is important to have a thorough medical history to identify if patients have predisposing conditions that may make one therapy more suitable than another. More research could be done on these medications to understand the benefits and risks of each thoroughly. In conclusion, hyperkalemia can be treated with a variety of different medications. Clinicians must be aware of these newer medications, which may have better selectivity for potassium excretion. These medications can aid in prompt treatment of patients who are at risk for or who suffer from hyperkalemia.
